# Are Healthcare Expenditures Related to Economic Growth in China? Bootstrap ARDL Approach

**DOI:** 10.3389/fpubh.2021.766091

**Published:** 2022-02-07

**Authors:** Yu-Cheng Chang, Tsangyao Chang, Mei-Chih Wang

**Affiliations:** ^1^Department of Leisure and Recreation Management, Asia University, Taichung, Taiwan; ^2^Department of Finance, Feng Chia University, Taichung, Taiwan; ^3^Chinese Social and Management Studies, Tung Hai University, Taichung, Taiwan

**Keywords:** aging ratio, bootstrap ARDL bound test, health care expenditures, economic growth, Granger causality test

## Abstract

This study attempts to investigate whether healthcare expenditures (HCE) are related to economic growth in China using a newly developed Bootstrap autoregressive distributed lag (ARDL) test for China over the period of 1990–2019. To avoid omitted variable bias, we use the ratio of the population of 65 years old over the total population (aging ratio) as a control variable. Empirical result indicates that no cointegration among these three variables. Granger causality test based on Bootstrap ARDL model demonstrates that one-way Granger causality running from HCE to aging ratio and from economic growth to both HCE and aging ratio. Empirical results have important policy implications for China understudy

## Introduction

Economists began to place greater emphasis on the role of human capital as a determinant of productivity and growth in the early 1990s. Since then, the importance of health and education in economic growth has received much attention (both theoretical and empirical), and a strong consensus has emerged in the last decade that human capital accumulation is an important determinant of economic growth. In addition to education, which constitutes one of the main resources of human capital, the health level of society is another important element. Therefore, it can be argued that there is a close relationship between the health level of society and its economic development.

This study contributes to current studies by investigating whether healthcare expenditures (HCE) lead to economic growth or vice versa, using bootstrap autoregressive distributed lag (ARDL) models, for China over the period 1990–2019. China provides an interesting arena to research for several reasons. First, China has some typical features of economic growth and has made remarkable economic progress over the last few decades with an annual average economic growth rate of 7–9% in the past two decades (1990–2015). Second, China's economy has become the second largest only next to the USA around the world since 2015. The overall economics in China in terms of total gross domestic product (GDP) will be sooner or later overpass that of the United States. Third, according to the UN's latest population data report that above 60 aged population will reach 12% in China. Although below the average of organisation for economic cooperation and development (OECD), the population aging problem is not prominent. However, the development trend of China's aging population will be accelerated since 2011 and in the next 30 years, China will become a comprehensive aging society. Until 2015, China's aging population will reach 0.248 billion about 17% of the total population. Based on a research report from the UN that the population of over 60 years old person will reach 0.5 billion in China, a number higher than the total population of the United States. Challenges of an aging population have become an important issue faced by the governments have taken a different mode of response. Finally, but not least, China started its open-door policy in the late 1970s, thus sufficient data are available for researchers to evaluate the effect of economic liberalization on economic phenomena.

The remainder of this study is organized as follows: Section II briefly describes previous literature, Section III presents the data used, Section IV describes the methodology used and the empirical findings and some policy implications are presented in Section V, and finally, Section IV concludes.

## Review of Literature

The role of healthcare spending in stimulating economic growth has been suggested in Mushkin's health-led growth hypothesis (1). According to this hypothesis, health is a type of capital; thus, investment in health can increase income and lead to overall economic growth. In fact, health can affect economic growth through its impact on human and physical capital accumulation (2). Since healthcare is a core component of human capital investment, rising national healthcare spending would tend to raise labor productivity, quality of life, and general welfare. Healthcare spending has also been credited for prolonging life expectancy and reducing morbidity and infant mortality rates (health outcomes) (3). Therefore, it can be stated that health is a significant form of human capital, and there is a close relationship between the health level of society and its economic development. However, with the development of a country's economy, its people tend to place greater value on the quality of life and, therefore, have a higher expectation of medical services—particularly in developed countries with higher national income (4). After World War II, there was an increase in the importance ascribed to the health sector in the national macroeconomic. Increasing HCE in a country cause increases in social security, tranquility, safety, and welfare, which leads to improved labor efficiency. HCE helps people with acute conditions to recover and return to work quickly. In general, healthier people can work harder and longer, and also think more clearly (5). Although HCE is ordinarily hypothesized to be a function of real per capita GDP, there are some reasons to suggest that this could be a bilateral relationship, as it can be reasoned that population health is an input to the macroeconomic production function (5).

There are some reasons why a bilateral relationship between HCE and real per capita income could exist. First, by definition, HCE is a function of resources available (income or wealth). Second, reverse causation—income as a function of HCE—also has a theoretical basis due to the fact that the latter is a determinant of (i) human capital and (ii) labor supply and productivity. HCE can be regarded as an investment in human capital (1, 6–8) and given that human capital is an “engine” of growth (9). Yang (10) surveys the developing countries finding that HCE and economic growth have significant effects due to different levels of human capital. When the level of human capital is low, HCE is significantly negatively correlated with economic growth. When the level of human capital is high, the positive economic impact of HCE is significantly enhanced. Similarly, rises in HCE make possible higher labor supply and productivity.

In this regard, there is a wide range of literature examining the relationship between HCE and economic growth, and this literature can be classified within different contexts in terms of its methodology, data, country group, period, and results. For example, Bhargava et al. (11) investigated the effects of health indicators on economic growth rates in the period 1965–1990 in developed and developing countries. In this panel data study, it was found that there is a positive but weak relationship between health and economic growth. Bloom et al. (12) extended production function models of economic growth, to account for work experience and health, for a panel of countries observed every 10 years from 1960 to 1990. Their result is that health has a positive and statistically significant effect on economic growth. Mayer (13) examined whether there is a Granger causality of HCE on income for 18 Latin American countries and found a strong causality of income on healthcare. Clemente et al. (14) analyzed the behavior of HCE for a number of the OECD countries. They adopted the cointegration approach and the results show that there is a long-term relationship between total HCE and GDP. However, the existence of cointegration is only shown when we admit the presence of some changes in the elasticity of the model. Bloom et al. (15) constructed a panel of countries observed every 5 years from 1960 to 1995 and they found that the estimated macroeconomic effects of health are positive and not significantly different from the microeconomic estimates. Erdil and Yetkiner (16) applied the Granger causality approach to panel data with fixed coefficients in order to determine the relationship between GDP and health expenditures per capita. The findings verify that the dominant type of causality is bidirectional, which cast doubt on the performance of ordinary least squares estimates in the literature. Moreover, one-way causality patterns are not similar for different income groups. One-way causality generally runs from income to health in lower- and middle-income countries, whereas the reverse holds true for higher-income countries. Lopreite and Zhu (17) used Bayesian-VAR (B-VAR) models to examine the aging index, life expectancy, economic growth, and health expenditure in China and the USA. They found that the aging index of the USA and China has a significant response to life expectancy and health expenditure per capita to GDP per capita, while population aging has a relatively strong response to health expenditure per capita in China.

Li and Huang (18) studied the relationship between per capita real GDP growth and the physical capital, human capital, and health investment in the production function. Panel data models were used in the estimation based on the provincial data from 1978 to 2005. The empirical evidence showed that both health and education have positive significant effects on economic growth. Çetin and Ecevit (19) examined the effect of health on economic growth using a panel data analysis for 15 OECD countries during the period from 1990 to 2006. They did not find any statistically significant relationship between health expenditures and economic growth. Wang (4) studied the international total HCE data of 31 countries from 1986 to 2007 to explore the causality between an increase in HCE and economic growth. Panel regression analysis and quantile regression analysis were used. The estimation of the panel regression reveals that health expenditure growth will stimulate economic growth; however, economic growth will reduce health expenditure growth. With regard to the estimation of quantile regression, in countries with a low level of growth, health expenditure growth will reduce economic growth. Mehrara et al. (20) examined the stationary and cointegration relationship between health expenditure and GDP based on the panel cointegration analysis for a sample of 13 the Middle East and North Africa (MENA) countries, using data for the period 1995–2005. The findings indicated that the share of health expenditures to GDP decreases with GDP. This implied that health care is not a luxury good in MENA countries. Amiri and Ventelou (5) investigated causality between GDP and HCE in OECD countries. They found that bidirectional Granger causality is predominant. Elmi and Sadeghi (2) investigated the causality and cointegration relationships between economic growth and HCE in developing countries from 1990 to 2009. Their findings indicated that income is an important factor across developing countries in the level and growth of HCE, in the long run. Additionally, the health-led growth hypothesis in developing countries is confirmed. Mehrara and Musai (21) studied the relationship between health expenditure and economic growth in Iran for the period 1970–2007, based on the ARDL approach. The study found a cointegrating relationship between real GDP, health expenditure, capital stock, oil revenues, and education, although among them health spending accounts for just a small part of the economic growth. They found that HCE did not make a significant marginal contribution to the economic growth in Iran. Ak (22) studied the existence of a long-term causality relationship between health expenditures, economic growth, and life expectancy at birth for the Turkish economy. As a result of the analysis, it was concluded that there is not a short-term relationship between the series, although there is a long-term relationship between health expenditures and economic growth. Odubunmi et al. (23) examined the relationship between HCE and economic growth in Nigeria for the period 1970–2009. They used the multivariate cointegration technique proposed by Johansen and found the existence of at least one cointegrating vector describing a long-run relationship between economic growth, foreign aid, health expenditure, total saving, and population. Bedir (24) use Toda and Yamamoto (25) approach to investigate HCE and economic growth nexus using data for 9 European and Middle East African countries (i.e., Czech Republic, Egypt, Greece, Hungary, Poland, Russia, South Africa, Turkey, and UAE) and 7 Asian countries (i.e., China, India, Indonesia, South Korea, Malaysia, the Philippines, and Thailand) and Bedir (24) found evidence in support of a two-way Granger causality for both the Czech R. and the Russian F. The evidence for the Egypt, Hungary, Korea R., South Africa, and the Philippines supports the health view over the income view and the evidence for Greece, Poland, South Africa, the UAE, China, Indonesia, and Korea R. supports the income view over the health view. The empirical results have indicated that income is an important factor for explaining the difference in HCE among countries. When economic growth occurs, the proportion of HCE in total GDP also increases. Conceptually, a healthy person can not only work more effectively and efficiently but also devote more time to productive activities. Since HCE is a core component of human capital investment, the rising trend of HCE would tend to raise labor productivity, quality of life, and general welfare. Healthcare spending has also been credited for prolonging life expectancy, reducing morbidity and infant mortality rates. Therefore, the growth in HCE has a positive influence on GDP. To sum up, economic growth causes an increase in HCE, and expenditure causes an increase in economic growth.

In the empirical literature, although there is no consensus on whether there is a direct relationship between the health status of countries and economic development, there is a consensus that higher social health status has a positive impact on the development of the country by increasing productivity. In conclusion, for some of the emerging market economies, it appears that increases in income level stimulate HCE.

Overall, for the past several decades, studies have been devoted to investigating what are the major determinants of economic growth in both developing countries and advanced countries. It is clear that health care policies often need to be adjusted in the short run term, but that overall health care strategies may also be targeted at longer-run objectives. In order to deal with these two issues, a newly developed Bootstrap ARDL approach was applied to explore the connection between HCE and economic growth in China.

## Data

**Figure 1:**
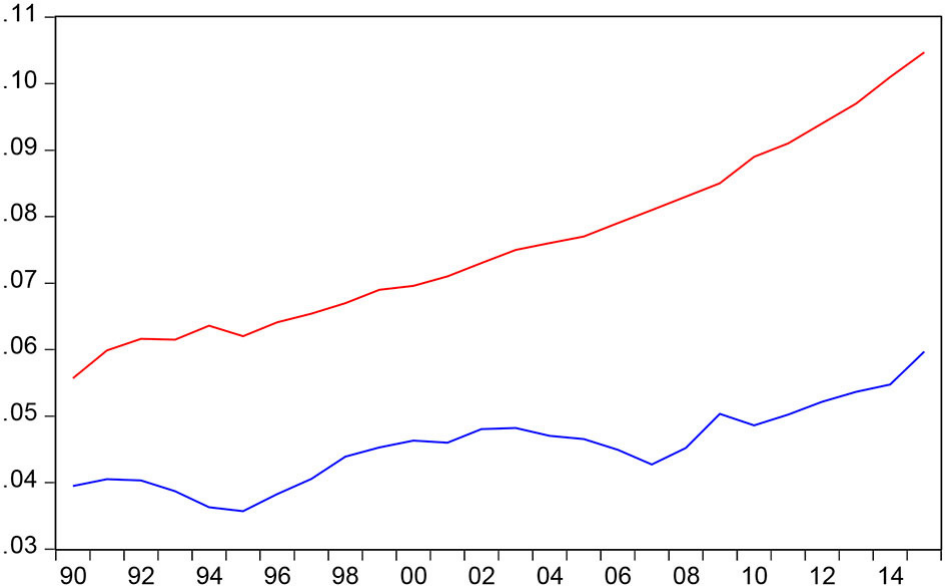
Plots of OLDR (65 years old ratio) and health expenditure over (HER GDP ratio).

This study aims to test whether there is causality between HCE and economic growth as well as whether HCE is a driving force for economic growth, particularly in China. To avoid omitted variable bias we also incorporate aging ratio into our Bootstrap ARDL model. The data are annual observations of GDP and total HCE in constant 2005 purchasing power parity (PPP) from 1990 to 2019. GDP, HCE, and aging ratio are taken from retrieved from the National Bureau of Statistics of China. Table 1 reports the summary statistics for the data series and Figure 1 plots the aging ratio (OLDR) and HCE as a share of GDP (HER) that we find OLDR continues to rise faster than HER in China. If we look at Figure 1 that we also find both HER and OLDR continue to increase substantially since 1990 and show a trending upward. The main goal of this study is to analyze the influence of HCE on economic growth. Because the improvements in health status will be worth the effort even if they turn out to have little effect on growth. The Jarque–Bera statistics indicate that both GDP and OLDR variables are normally distributed and HE is normally distributed.

**Table 1:** Data description.

**Variable**	**Mean**	**Median**	**Maximum**	**Minimum**	**Skewness**	**Kurtosis**	**Jarque-bera (***P***-value)**
GDP	225574.4	128528.4	686449.6	18923.30	0.9563	2.5581	4.1742
Age	0.0760	0.0740	0.1047	0.0557	0.5352	2.2486	1.8531
Health	11299.89	6187.07	40974.64	747.39	1.1912	3.2145	6.1983**

^***, **^, and ^*^ indicate significance at the 1, 5, and 10% levels, respectively.

## Methodology

### Bootstrap ARDL Test

Based on Pesaran et al. (26) that we can write our ARDL Bound model as follows:


(1) $$\begin{array}{l} \begin{array}{ccc} \Delta Y_{t} & = & c+\alpha Y_{t-1}+\beta X_{t-1}+\overset{p-1}{\underset{i=1}{\sum^{\text{​}}}}\theta \Delta Y_{t-i}+\overset{p-1}{\underset{i=1}{\sum^{\text{​}}}}\delta X_{t-i} \end{array}\\ \begin{array}{cc} + & \overset{q}{\underset{j=1}{\sum^{\text{​}}}}\eta D_{t,j}+\varepsilon_{t} \end{array} \end{array}$$

Equation (1) requires no feedback from Y to X. This means that we cannot allow two or more variables to be (weakly) endogeneous and this violates the assumptions underlying the distributions of the test statistics presented by Pesaran et al. (26). It assumes weak exogeneity of the regressors. In the long run, these regressions are not affected by the dependent variable, but this does not rule out the existence of a cointegration relationship between the regressions, nor does it assume that there is no (shortterm) Granger causality between the dependent variable. A lot of researchers from previous studies ignore this assumption in the empirical implications of the ARDL bounds test. According to Pesaran et al. (26), cointegration test requires *F*-test or *t*-test for testing the following hypothesis:


$$\begin{array}{l} H_{0}:\alpha =\beta =0orH_{0}:\alpha =0 \end{array}$$

McNown et al. (27) suggest that by adding a *t*-test *H*_0_:β = 0 to complement the existing *F*- and *t*-tests for cointegration proposed by Pesaran et al. (26). The use of all three tests is necessary to distinguish between cases of cointegration, noncointegration, and degenerate cases defined by Pesaran et al. (26). Based on McNown et al. (27) that we can define the two degenerate cases as follows:

**Degenerate case #1** occurs when the *F*-test and the *t*-test on the lagged independent variable are significant, but the *t*-test on the lagged dependent variable is insignificant.
**Degenerate case #2** occurs when the *F*-test and the *t*-test on the lagged dependent are significant, but the lagged independent variables are not significant.

Pesaran et al. (26) present critical values for case #2, but not for case #1. To rule out degenerate case #1, the integration order for the dependent variable must be I(1). However, unit root tests are notorious for having low power (28). The Bootstrap ARDL test tackles this problem through the additional test on the coefficients of the lagged independent variables. The advantage of the Bootstrap ARDL Bound Test is that there is evidence that the endogeneity problem has only minor effects on the size and power properties of the ARDL Bounds testing framework using the asymptotic critical values from the Monte Carlo simulations. In addition, if the resampling procedure is applied appropriately, the Bootstrap test performs better than the asymptotic test in the ARDL Bounds test based on size and power properties. Furthermore, the Bootstrap procedure has the additional advantage of eliminating the possibility of inconclusive inferences. Finally, McNown et al. (27) also present an extension of the ARDL testing framework for the alternative degenerate case, with critical values generated by the Bootstrap procedure. Therefore, the proposed Bootstrap ARDL test provides a better insight into the cointegration status of the series in the model.

### Granger Causality Test Based on Bootstrap ARDL Model

The direction of the short-run causal relationship will be determined by standard Granger-causality tests. If no cointegration is found between y and x when y is the dependent variable, then the Granger causality test for x ≥ y should include the lagged differences on x only, that is, we test whether δ>0. However, if cointegration exists among the variables, then this means the dependent and the independent variables form a stationary linear combination. As a result, the lagged levels can be treated as I(0). In this case, the Granger-causality test for x ≥ y should include the lagged differences on x and the lagged level of x, i.e., test whether β>0 and δ = 0. Of course, that we can also extend the Equation (1) to the 3-variable case, refer to the following model:


(2) $$\begin{array}{l} \begin{array}{c} \Delta Y_{t}=c+\alpha Y_{t-1}+\beta X_{t-1}+\gamma Z_{t-1}+\overset{p-1}{\underset{i=1}{\sum^{\text{​}}}}\theta \Delta Y_{t-i}+\overset{p-1}{\underset{i=1}{\sum^{\text{​}}}}\delta \Delta X_{t-i}+ \end{array}\\ \begin{array}{c} \overset{p-1}{\underset{i=1}{\sum^{\text{​}}}}\omega Z_{t-i}+\overset{j}{\underset{j=1}{\sum^{\text{​}}}}\eta D_{t,j}+\varepsilon_{t} \end{array} \end{array}$$

In this case, the Granger-causality test for x ≥ y should include the lagged differences on x and the lagged level of x, i.e., test whether β>0 andδ = 0. For z = > y should include the lagged differences on z and the lagged level of z, i.e., test whether γ>0andω = 0 (if they are cointegrated).

## Empirical Results and Policy Implications

In this study, we employ the Bootstrap ARDL Bound Test of cointegration technique advanced by McNown et al. (27) to test for a long-run relationship between HCE and economic growth in China over 1990–2019. The Bootstrap ARDL approach to cointegration testing has several interesting characteristics. First, it performs better to small samples compared to alternative multivariate cointegration procedures (29). Second, it does not require the restrictive assumption that all series are integrated of the same order allowing for the inclusion of both *I*(0) and *I*(1) [but not *I*(2)] time series in a long-run relationship; the latter provides flexibility and also avoids potential “pretest bias” that means, the specification of a long-run model on the basis of I(1) variables only (26).

### Results From the Unit Root Test

Because the Bootstrap ARDL Bound Test approach does not require the restrictive assumption that all series are integrated of the same order, thus allowing for the inclusion of both *I*(0) and *I*(1) time series in a long-run relationship, however, the presence of *I*(2) variables turns the computed *F*_*PSS*_ statistic invalid (26). Therefore, we need to first go for several conventional unit root tests such as the augmented Dickey-Fuller test, Phillips-Perron (PP) (30), and Kwiatkowski-Phillips-Schmidt-Shin test (KPSS) (31). Table 2 reports the results from several conventional unit root tests which all suggest that these three variables employed are all nonstationary in levels, while they turn stationary in first differences.

**Table 2:** Univariate unit root tests.

	**Level**			**First differences**		
	**ADF**	**PP**	**KPSS**	**ADF**	**PP**	**KPSS**
GDP	−2.648629[3]	0.440807[2]	0.197280[3]**	−2.619193[0]	−2.588210[5]	0.132870[2]*
Age	1.126139 [1]	1.211325 [2]	0.189014[3]**	−1.609022[1]	−4.176956[3]***	0.499507 [2]**
Health	5.140937[0]	5.140937 [0]	0.195521[3]**	−2.045784 [4]	−1.193126[5]	0.191995[3]**

^***, **^, and ^*^ indicate the null hypothesis is rejected at the 1, 5, and 10% levels, respectively. The number in brackets indicates the lag order selected based on the Schwarz information criterion. The number in the parenthesis indicates the truncation for the Bartlett Kernel, as suggested by the Newey–West test (1987).

### Results From Bootstrap ARDL Test—Cointegration Test

Because we have established that all variables are integrated of one [or I (1)], we proceed to test for cointegration by employing the Bootstrap ARDL test approach. The selection of the optimal Bootstrap ARDL specifications is selected based on the Schwarz information criterion which is asymptotically consistent for the lag length and is favored by Pesaran and Shin (32). The selection of the optimal nonlinear ARDL specifications is based on a general-to-specific approach, starting with *max p* = *max q* = 4 and dropping all the insignificant lags using a 5% decision rule. The *F*_*PSS*_ statistics of the Bootstrap ARDL approach being reported in Table 3 indicate strong evidence in favor of the nonexistence of a long-run cointegrating relationship among GDP, HCE, and aging ratio in China. Therefore, we proceed to test the Granger causality test based on our Bootstrap ARDL model in difference.

**Table 3:** Cointegration results using Bootstrap autoregressive distributed lag (ARDL) bound test.

**Variable**	**DV|IV**	**Dummy variables**	**F**	F*_	**T_dep_**	T*_dep_	**F_indep_**	F*_indep_	**Result**
GDP	GDP| age,health	D95 d04 d10	**4.501**	3.737	−1.828	−2.170	2.346	3.857	Degenerate #1
Age	age| GDP,health	d99 d07 d12	**7.653**	4.478	2.715	−1.889	1.437	4.944	Degenerate #1
Health (1990–2015)	health | GDP,age	D95 d03 d09	2.112	4.492	−1.314	−2.796	0.794	4.861	No-cointegration

[.] is optimal lag order based on Akaike Information Criterion (AIC). F is the F-statistic for the coefficients of yt−1, x t−1, and zt−1; Tdep denotes the t-statistics for the dependent variable, Tindep denotes the t-statistics for the independent variable. F^*^, T_dep, and T_indep are the critical values at a 5% significance level, generated from the bootstrap program. Dummy variables are to capture any economic shocks. D03 means 1 for the year 2003, other years are 0.

### Granger Causality Test Results Based on Bootstrap ARDL Model and Policy Implications

Table 4 reports Granger causality test results based on the Bootstrap ARDL model.

**Table 4:** ARDL Granger-causality analysis.

	**ΔGDP equation:**	**ΔAge equation:**	**Δhealth equation:**
	**ΔAge, Δhealth**	**ΔGDP, Δhealth**,	**ΔGDP, ΔAge**
	**F- statistics**	**F- statistics**	**F- statistics**
	**(*p*-value)(Sign)**	**(*p*-value)(Sign)**	**(*p*-value)(Sign)**
GDP[2]	n.a.	7.075**(0.012)(-)	9.737***(0.004)(+)
Age[2]	0.671(0.533)(+)	n.a.	0.3696(0.7001)(+)
Health[2]	2.170(0.164)(–)	3.44*(0.072(+)	n.a.

*Value in [.] is lag order, and (.) is p value and sign for the coefficients. Bold values refer to the case of cointegration and the causality test involved its lagged level and differenced variables. Those values not in bold refer to the case of no-cointegration and its causality test involved only lagged differenced variables*.

^***^, ^**^, and ^*^ denote significance at 1, 5, and 10% levels, respectively.

**Figure 2:**
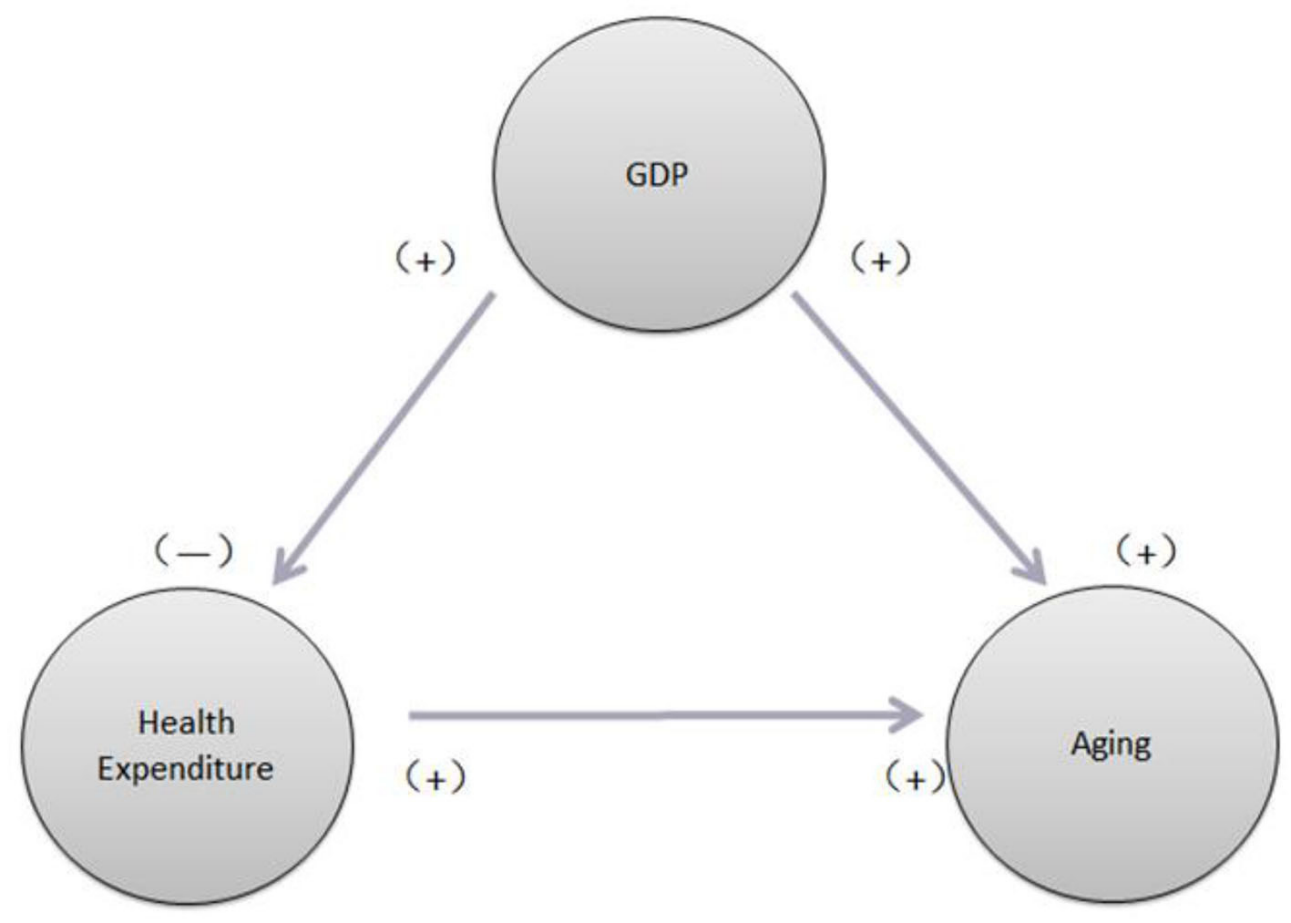
Causal links among healthcare expenditure (HCE), aging ratio (OLDR) gross domestic product (GDP) (economic growth).

From Table 4, we can see a one-way Granger causality running from HCE to aging ratio and from economic growth to both HCE and aging ratio. If we look at the sign of all coefficients of the independent variables and we find that economic growth has significantly positive effects on both HCE and aging ratio and HCE also affects the aging ratio positively and significantly. Interesting is that we also find both the aging ratio (positively) and HCE (negatively) affect economic growth, respectively but not significant. Based on empirical results that we find economic growth can boom up both HCE and increase people's life expectancy (means more and more elder person) and the more HCEs the more health people (elder people). As we know aging become a very important threat for China, especially for these several years. Based on statistic reports, there are more than 264 million elder people at the end of 2020, around 18.7% of the total population. This aging problem not only hurts economic development but also damages the healthcare system (expenditures go up). Our empirical results have important policy implications for the government of China conducting health care policy to sustain its economic growth. Figure 2 demonstrates the causal relationship among these three variables [i.e., economic growth (GDP), HCE, and aging]. This figure further confirms our empirical findings.

## Conclusion

In this study, we attempt to investigate the impact of HCE s on economic growth (or GDP) in China using a newly developed Bootstrap ARDL model over the period of 1990–2019. To avoid omitted variable bias we use the ratio of the population of 65 years old over total population (aging ratio) as a control variable Empirical results indicate no long-run relationship among these three variables and the Granger causality test based on Bootstrap ARDL model indicates a one-way Granger causality running from HCE to aging ratio and from economic growth to both HCEs and aging ratio. Our study has important policy implications for China under study.

## Data Availability Statement

The raw data supporting the conclusions of this article will be made available by the authors, without undue reservation.

## Author Contributions

Y-CC is responsible for organizing the progress of the paper, final methods, and comprehensive management of the structure, and content of the article. TC is responsible for data collection and analysis, part of the revision, and finalization. M-CW is responsible for guiding the topic selection, research manuscript writing, and submission. All authors contributed to the article and approved the submitted version.

## Conflict of Interest

The authors declare that the research was conducted in the absence of any commercial or financial relationships that could be construed as a potential conflict of interest.

## Publisher's Note

All claims expressed in this article are solely those of the authors and do not necessarily represent those of their affiliated organizations, or those of the publisher, the editors and the reviewers. Any product that may be evaluated in this article, or claim that may be made by its manufacturer, is not guaranteed or endorsed by the publisher.
